# DNA Methylation Profiles in a Group of Workers Occupationally Exposed to Nanoparticles

**DOI:** 10.3390/ijms21072420

**Published:** 2020-03-31

**Authors:** Andrea Rossnerova, Katerina Honkova, Daniela Pelclova, Vladimir Zdimal, Jaroslav A. Hubacek, Irena Chvojkova, Kristyna Vrbova, Pavel Rossner, Jan Topinka, Stepanka Vlckova, Zdenka Fenclova, Lucie Lischkova, Pavlina Klusackova, Jaroslav Schwarz, Jakub Ondracek, Lucie Ondrackova, Martin Kostejn, Jiri Klema, Stepanka Dvorackova

**Affiliations:** 1 Department of Genetic Toxicology and Epigenetics, Institute of Experimental Medicine CAS, Videnska 1083, 142 20 Prague 4, Czech Republic; katerina.honkova@iem.cas.cz (K.H.); irena.chvojkova@iem.cas.cz (I.C.); jan.topinka@iem.cas.cz (J.T.); 2 Department of Occupational Medicine, First Faculty of Medicine, Charles University in Prague and General University Hospital in Prague, Na Bojisti 1, 120 00 Prague 2, Czech Republic; daniela@pelclova.cz (D.P.); Stepanka.Vlckova@vfn.cz (S.V.); zdenka.fenclova@lf1.cuni.cz (Z.F.); Lucie.Lischkova@vfn.cz (L.L.); pavlina.klusackova@vfn.cz (P.K.); 3 Laboratory of Aerosol Chemistry and Physics, Institute of Chemical Process Fundamentals CAS, Rozvojova 1, 165 02 Prague 6, Czech Republic; zdimal@icpf.cas.cz (V.Z.); schwarz@icpf.cas.cz (J.S.); ondracek@icpf.cas.cz (J.O.); ondrackova@icpf.cas.cz (L.O.); kostejn@icpf.cas.cz (M.K.); 4 Center for Experimental Medicine, Institute for Clinical and Experimental Medicine, Videnska 1958/9, 140 21 Prague 4, Czech Republic; jahb@ikem.cz; 5 Department of Nanotoxicology and Molecular Epidemiology, Institute of Experimental Medicine CAS, Videnska 1083, 142 20 Prague 4, Czech Republic; kristyna.vrbova@iem.cas.cz (K.V.); pavel.rossner@iem.cas.cz (P.R.); 6 Department of Computer Science, Czech Technical University in Prague, Karlovo namesti 13, 121 35 Prague 2, Czech Republic; klema@fel.cvut.cz; 7 Department of Machining and Assembly, Department of Engineering Technology, Department of Material Science, Faculty of Mechanical Engineering, Technical University in Liberec, Studentska 1402/2 Liberec, Czech Republic; stepanka.dvorackova@tul.cz

**Keywords:** 850K microarray, CpG sites, DNA methylation, epigenetic adaptation, human, nanoparticles, occupational exposure

## Abstract

The risk of exposure to nanoparticles (NPs) has rapidly increased during the last decade due to the vast use of nanomaterials (NMs) in many areas of human life. Despite this fact, human biomonitoring studies focused on the effect of NP exposure on DNA alterations are still rare. Furthermore, there are virtually no epigenetic data available. In this study, we investigated global and gene-specific DNA methylation profiles in a group of 20 long-term (mean 14.5 years) exposed, nanocomposite, research workers and in 20 controls. Both groups were sampled twice/day (pre-shift and post-shift) in September 2018. We applied Infinium Methylation Assay, using the Infinium MethylationEPIC BeadChips with more than 850,000 CpG loci, for identification of the DNA methylation pattern in the studied groups. Aerosol exposure monitoring, including two nanosized fractions, was also performed as proof of acute NP exposure. The obtained array data showed significant differences in methylation between the exposed and control groups related to long-term exposure, specifically 341 CpG loci were hypomethylated and 364 hypermethylated. The most significant CpG differences were mainly detected in genes involved in lipid metabolism, the immune system, lung functions, signaling pathways, cancer development and xenobiotic detoxification. In contrast, short-term acute NP exposure was not accompanied by DNA methylation changes. In summary, long-term (years) exposure to NP is associated with DNA epigenetic alterations.

## 1. Introduction

The production and wide use of many different nanomaterials (NMs) during the last decade has raised questions regarding their safety for human health and especially for genetic information. Due to the size range of nanoparticles (NPs), i.e., structures between 1 and 100 nm in one or more external dimensions [1], there is a valid risk of their entry to cells and even possible direct or indirect negative effects for DNA, with width variance between 2 nm for relaxed double helix and 1400 nm for two chromatid fully coiled chromosomes including histones. Aside from the ability of NPs to enter the organism and the cells, it is also important to highlight their high diversity in size, shape, dimensions of aggregates, chemical composition or their charge, as well as many, mostly unknown interactions in various environments including the microenvironment of the cells. All these facts raise various issues for toxicologists to address.

To date, a relatively high number of studies focused on the effect of exposure to NPs, using various cell line in vitro models, have been published. A wide spectrum of biomarkers has been used, and also a wide range of results (from negative to positive effects) have been obtained due to the high diversity of NPs. Surprisingly, studies focusing on DNA alterations including methylation changes, which play an important role in the reprogramming of gene expression intensity and the adaptation ability of an organism with long-term consequences, are still less common despite growing interest over the last few years.

Differences in the level of 5-methyl-cytosine (5-mC) were studied in several cell lines after exposure to various NPs, but the results substantially differed depending on the selected NP, cell line and methodological approach. One of the first studies, which was published in 2010, investigated the effect of 15 nm silicone dioxide (SiO_2_) NPs in human keratinocytes (HaCaT cells). The results showed global DNA hypomethylation with the downregulation of two DNA methyltransferase genes, *DNMT1* and *DNMT3a*. The authors also highlighted the possible long-term effects of epigenetic changes [2]. The same trend was also reported for the effects of titanium dioxide (TiO_2_) and zinc oxide (ZnO) in lung fibroblast MRC5 cells [3]. Another study analyzed the effect of various carbon NPs including single- and multi-walled nanotubes (SWCNTs and MWCNTs) in human A549 lung cancer cells. Hypermethylation, following exposure to all carbon NPs, was reported for this hypotriploid cancer cell line [4]. The epigenetic effects of exposure to MWCNTs and SWCNTs were also studied in bronchial epithelial cells (16HBE14o-), where no global DNA methylation alteration on 5-mC was observed, but a detailed array screening revealed hypo- and/or hypermethylation, in particular on CpG sites [5]. The same array analysis also revealed minor effects of TiO_2_ and MWCNTs exposure in human bronchial epithelial lung cells (BEAS-2B), where the altered CpG sites were mainly located in low-density regions and very frequently on the X chromosome [6]. The effects of industry-relevant engineered NM (printer-emitted engineered NP, mild steel welding fumes, copper oxide (CuO) and TiO_2_) on the epigenome of three cell lines was the object of a further study. The authors concluded that short-term exposure to engineered NM modestly affected DNA methylation within the most abundant transposable elements [7]. A detailed DNA methylation profile of DNA methyltransferase genes in breast cancer cell line MCF-7 after treatment with maghemite nanoparticles revealed epigenetic changes, even though the overall percentage of DNA methylation was not affected [8], which in contrast to altered global methylation by the same nanomaterial but in human submandibular gland cells, which was analyzed in a later study [9]. No significant differences in the DNA methylation status of inflammatory and apoptosis response genes of human liver cancer cells (HepG2) was found after silver, gold and superparamagnetic iron nanoparticle (SPION) exposure [10].

Most of the in vivo mammalian studies were only recently published. They provide new and important knowledge on related NP exposure lasting for up to 10 weeks. The effects of Au NPs, SWCNTs and MWCNTs in BALB/c mice on DNA methylation revealed a deregulation in the genes of immune pathways and differences, in effect related to various exposure doses [11]. Another study analyzed the effect of inhalation of TiO_2_ NPs in mice differing by age. The results showed that young mice (5 weeks) had more frequently altered methylation after exposure than adult animals (10 weeks) [12]. A further recent study analyzed the effects of 4 week diet supplementation with Cu NPs in rats. The exposure not only caused reduced protein oxidation and nitration, but it also affected DNA oxidation and methylation [13].

To date, only one study has investigated DNA methylation alterations in humans. In addition to global methylation in white blood cells, oxidative DNA damage, and lipid peroxidation were analyzed in workers who were occupationally exposed to metal oxide nanomaterials (TiO_2_, SiO_2_ and indium tin oxide (ITO)). Global DNA hypomethylation was detected in a subgroup of ITO exposed workers, along with increased levels of oxidative damage markers [14].

It is also important to mention the methodological approaches used in DNA methylation studies, as differences in the information output can affect the interpretation of the results. Current knowledge has allowed the application of a wide spectrum of methods, some of which provide quantitative information on global DNA methylation, while others generate qualitative data about site-/gene-/region-/whole genome-specific DNA methylation [15,16,17,18,19]. Most of the above-mentioned studies conducted identification of global DNA methylation changes, which represent a cheaper solution but has limited interpretation due to potentially hidden changes. This is particularly evident when the levels of hypermethylated and hypomethylated CpG loci are balanced, as, for example, in a study focused on the effect of exposure to SWCNTs and MWCNTs. In this study, detailed array screening using Human Methylation 450K BeadChip revealed specific differences [5]. This state-of-the-art methylation assay is whole-genome bisulfite sequencing (WGBS), which provides complete information on site-specific differences. However, this solution is too costly to be accessible for larger numbers of samples. An alternative solution is the application of array chips that provide information about DNA methylation status in specific CpG sites. Today, 866,895 selected CpG loci distributed throughout the whole human genome can be analyzed per sample [19]. As yet, this solution has not been used for the investigation of epigenome alterations caused by NP exposure.

In this study, we aimed to fill the gaps in knowledge related to possible DNA alterations in a human population exposed long-term and short-term to NPs. We continued with our research focused on biomonitoring researchers exposed to NP during the nanocomposite producing processes (welding, machining). We had already analyzed the markers of oxidative stress, cytogenetic alterations (levels of total and centromere positive and negative micronuclei (MN)), leukocyte telomere length, as well as DNA damage assessed by comet assay [20,21,22,23,24,25]. The results obtained by micronucleus assay showed a lack of long-term (years) exposure effects in contrast to the evident effects of short-term exposure. Similar trends were also observed in relation to air pollution exposure in our previous studies where we revealed differences in the epigenetic pattern (analyzed by 27K BeadChips) associated with particular locations. These results were interpreted as epigenetic adaptation to the environment [26,27]. We also presume that this phenomenon has general validity in relation to the long-term effects of various stressors. We intended to verify our theory and, thus, investigated the possible differences in DNA methylation patterns by 850K BeadChips in the group of nanocomposite research workers and unexposed controls. We also hypothesized that if our theory is correct, the overall range of differently methylated CpG sites caused by NP exposure will be lower than in the case of air pollution due to the different (shorter) time range of exposure during a lifetime.

## 2. Results

### 2.1. Exposure Online Monitoring by Scanning Mobility Particle Sizer (SMPS) and Aerodynamic Particle Sizer (APS) and Proportions of Particulate Matter (PM) Fractions

Exposure to five PM fractions (ranging from <25 nm to 10 µm) including two nano-fractions (<25 nm and 25–100 nm) was measured by on-line SMPA and APS monitoring (details in Section 4.2) in two workshops and two background spaces. Activities in workshop 1 included metal active gas (MAG) welding technology on mild steel S355J2 with wt % content: Fe (97.39%), Mn (1.7%), Si (0.6%), C (0.24%), P (0.035%) and S (0.035%). In workshop 2, machining (grinding and milling) of one type of sample containing epoxide resin with up to 20% SiO_2_ including nano-fraction as a filler was carried out. The first background space was located in the basement (same as workshop 1), the second one was located on the ground floor (same as workshop 2).

**Figure 1:**
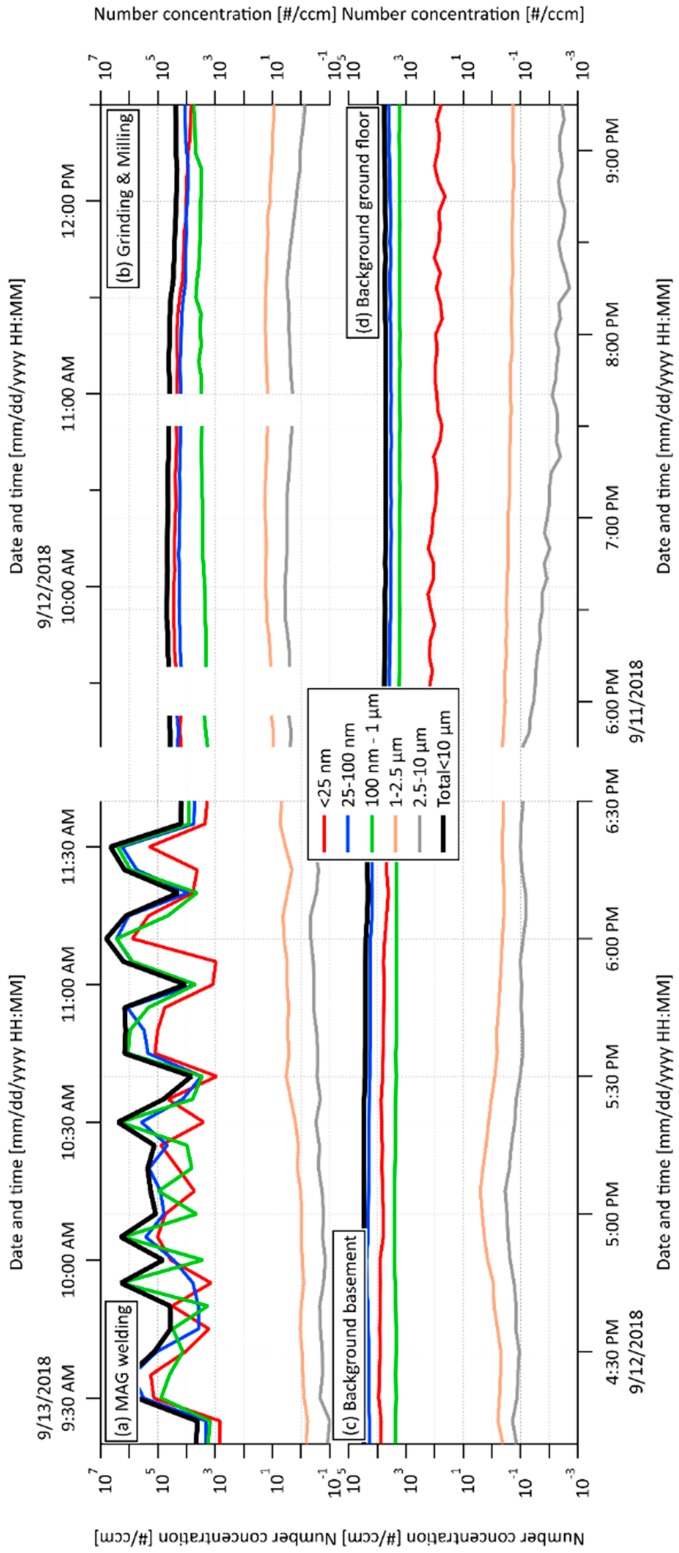
Scanning Mobility Particle Sizer (SMPS) + Aerodynamic Particle Sizer (APS) concentration in wider size bins related to (**a**) metal active gas (MAG) welding process in workshop 1; (**b**) grinding and milling processes in workshop 2; (**c**) background, basement; (**d**) background, ground floor.

The SMPS+APS number concentration in wider size bins for individual spaces and their time changes are shown in Figure 1. The total number concentrations of all five PM fractions (nano to 10 µm) per cm^3^ measured by this on-line monitoring are presented in Table 1. Periodicity vs. continual production of PM related to individual working processes is clearly visible from Figure 1a,b. In general, the obtained results showed the majority of the concentrations of PM nano-fractions was 25–100 nm in all monitored spaces (including both background spaces), with substantially increased total concentration related to grinding and milling processes (Table 1). The highest level of the smallest PM fraction (<25 nm) was detected in workshop 2. Moreover, the total concentrations of both nano-fractions (26,100 NP per cm^3^) exceeded 4.73 x the level detected in workshop 1, indicating that grinding and milling processes represent a greater risk of the production of NP in comparison with the MAG welding process. Nevertheless, the concentration of the smallest nano-fraction was still increased in workshop 1 in comparison with both background spaces (2.93 x and 18.2 x higher than background basement and background ground floor, respectively). Interestingly, the concentration of the PM fraction from 25 to 100 nm in the background-basement was higher than that in workshop 1 (MAG welding), while the concentration of the PM fraction <25 nm in the background-basement was lower than that in workshop 1 on the same floor. This can be explained by the accumulation and agglomeration of the smallest particles produced during welding.

### 2.2. Global DNA Methylation

In this study, we investigated the possible alterations in DNA methylation in the genome of nanocomposite research workers and matched controls using two methodological approaches: (i) quantitative analysis, which provides information about global DNA methylation (2.2), and (ii) a qualitative approach via microarray analysis of more than 850,000 CpG loci distributed in all genes including individual regions, as well as intergenic parts of human genome (2.3).

**Figure 2:**
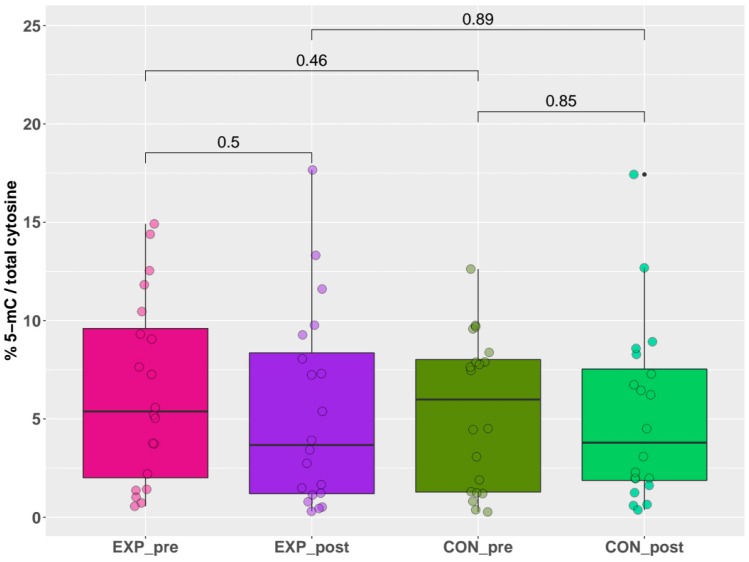
Pre-shift and post-shift levels of % 5-mC in exposed and control groups.

The results of global DNA methylation, obtained by the indirect ELISA technique, are summarized in Figure 2. These results uniformly showed no significant differences (*p* > 0.05) in the % of 5-mC for any comparison between individual groups (exposed pre-shift x exposed post-shift (short-term acute exposure), controls pre-shift x controls post-shift, exposed pre-shift x controls pre-shift (long-term chronic exposure), and exposed post-shift x controls post-shift). Briefly, no effect of either acute or chronic exposure was detected by this quantitative method.

### 2.3. Genome-Wide DNA Methylation Microarray Analysis

**Figure 3:**
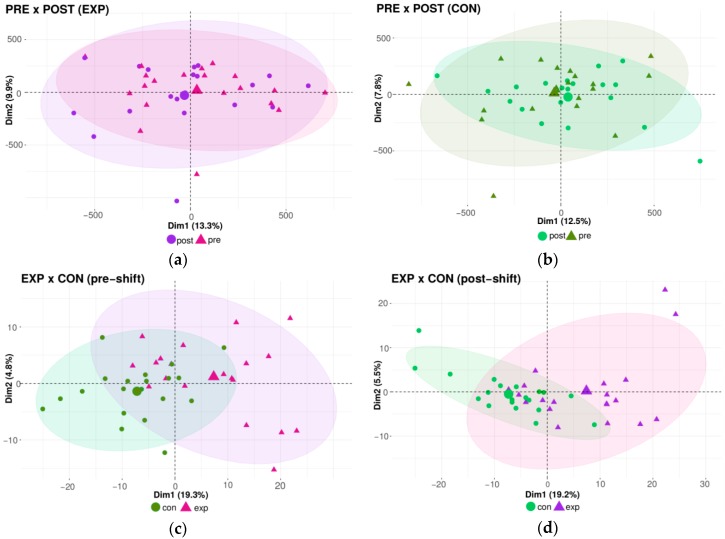
The impact of the nanoparticle exposure on the DNA methylation pattern; analyzed by PCA. (**a**) Comparison between exposed pre-shift and exposed post-shift groups. (**b**) Comparison between control pre-shift and control post-shift groups. (**c**) Comparison between exposed pre-shift and control pre-shift groups. (**d**) Comparison between exposed post-shift and control post-shift groups.

Using the qualitative approach via microarray analysis, we first conducted the same comparison between groups as in the case of the global methylation analysis. The results of the DNA methylation pattern similarities, analyzed by principal component analysis (PCA), are shown in Figure 3a–d). The outcomes indicate no differences in the DNA methylation pattern related to acute, daily shift exposure between the pre-shift and post-shift samples obtained from both the exposed and control groups (Figure 3a,b). In contrast, the effect of chronic long-term (years) exposure was manifested by the clustering of exposed and controls groups (Figure 3c,d). In summary, no effect of acute exposure was detected by this qualitative method, in contrast to a trend of changes to the DNA methylation pattern after chronic exposure.

A detailed statistical analysis revealed a total of 705 CpG loci with significant differences in methylation between the exposed subjects and controls (adjusted *p* value < 0.05, range: 0.0000029–0.049937). From these loci, 341 and 364 sites were hypomethylated and hypermethylated in the exposed group in comparison to the control, respectively. Moreover, 64.8% of significant results were annotated to particular genes including their regions: 200 bp or 1500 bp blocks upstream of the transcription start site (TSS200 or TSS1500) in the promoter part followed by 5′UTR, first exon, gene body and 3’UTR regions. Additional data analysis revealed no uniformed distribution across individual autosomes. Particularly, chromosomes #10, #1, #2 and #19 with 69, 68, 58 and 51 significant differences in CpG loci methylation, respectively, were the most frequently affected. In contrast, chromosomes #21, #14 and #22, #18 with 7, 14 and 14, 15 significant differences in CpG loci methylation, respectively, were the least frequently affected.

**Figure 4:**
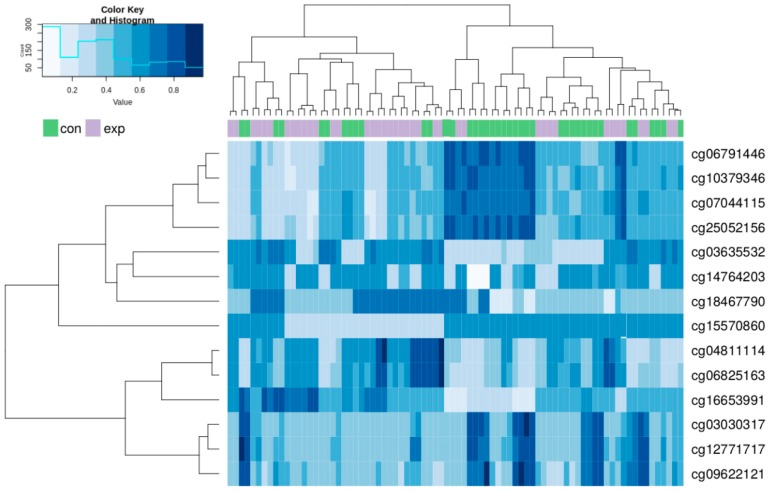
Hierarchical clustering analysis of the most differentially methylated CpG loci in the exposed and control group. Columns represent individual samples (exposed, purple; controls, green); methylation intensities in individual CpG sites identified by cg numbers are in rows.

**Figure A1:**
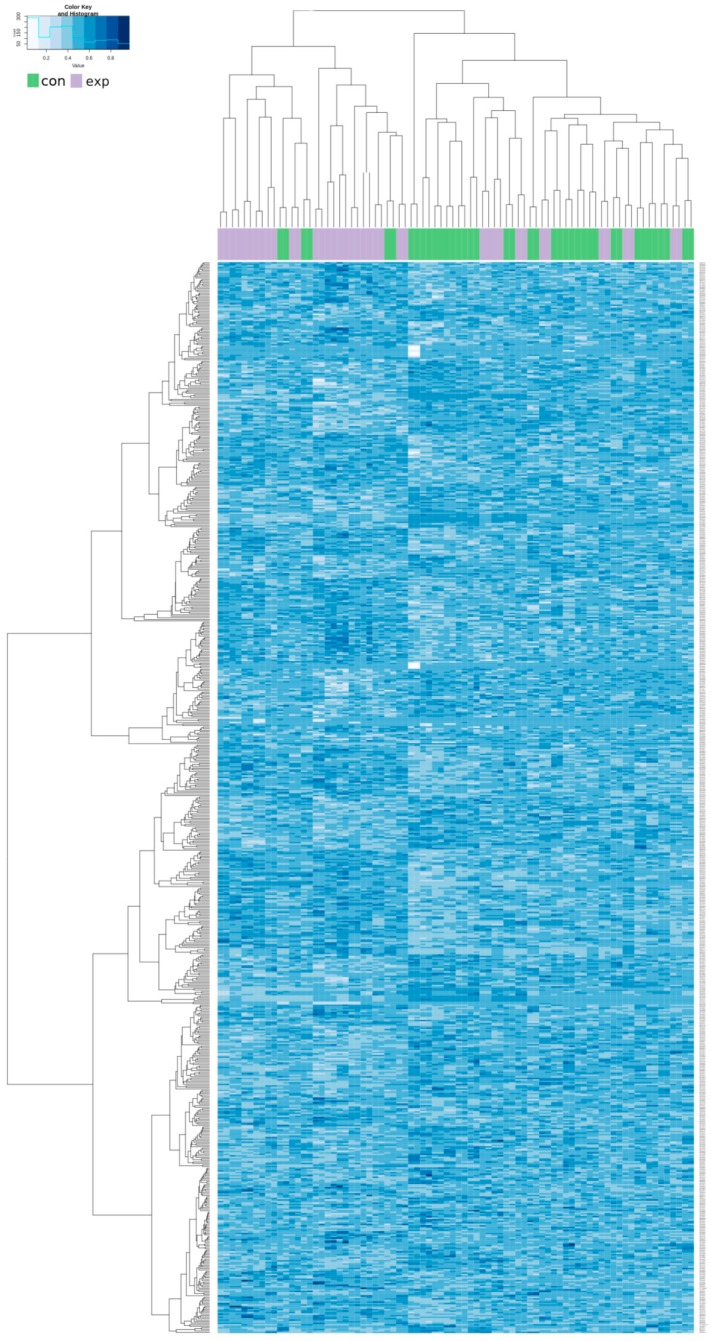
Hierarchical clustering analysis of all 705 differentially methylated CpG loci in exposed und control groups. Columns represent individual samples (exposed, purple; controls, green); methylation intensity in individual CpG sites are in rows.

A hierarchical clustering analysis was done for all of the above-described 705 CpG loci in both groups. The results showed a trend to separation of the exposed and control group. These synoptic results are presented in Appendix A as Figure A1. After restriction of the clustering analysis to the results with log fold change (FC) > 1.5 and log FC < −1.5, we obtained the 14 most significant results (8 hypomethylated and 6 hypermethylated). The results of the new hierarchical clustering analysis including individual cg numbers of individual CpG loci for all participants of both the exposed and control group showed more details (data presented in Figure 4).

An overview of information related to the 14 most differentially methylated CpG loci (highlighted in bold) in the exposed group when compared with the controls, as well as all other significant results in the same gene, is presented in Table 2. Additionally, an overview of information related to other significant results with three or more differently methylated CpG in the same gene is presented in Table 3. Both tables summarize data related to chromosome location, cg number, location in gene or outside of gene in intergenic regions, information about location of the CpG relative to the CpG islands (open sea, island, N/S shore, N/S shelf) and relevance or phenotype details related to individual genes obtained from GeneCards^®^ in the humane gene database https://www.genecards.org/.

The data presented in Table 2 indicate that (i) the majority of highly significant CpG in chromosome #10 (almost 36%) are prevalently located in *FGFR2* gene with a total of 23 significantly changed cg with prevalence (74%) of hypomethylation; (ii) in total, 5 protein coding genes (*LGR6, RADIL, FGFR2, TMEM9B, FCGBP*), 1 RNA coding gene (*HCG27*) and 2 intergenic cg located out of genes in two chromosomes were among the most significant results; (iii) significant CpG loci were generally located in parts with various CpG density, mostly with low (open sea) but also with high density (two cg of CpG island of the gene *FCGBP*); (iv) main differences in CpG methylation levels between the exposed subjects and controls were detected in genes related to signaling pathways including cytokines involved in the immune system, in genes involved in lung functions, asthma, various cancers, blood cell count and lipid metabolisms including the impact on body mass index (BMI).

Additional significant results presented in Table 3 show (i) 12 additional protein coding genes with 3 or more differentially methylated CpG loci in the same gene (*LOC100996579, TMEM18, SHISA3, UGT2B15 and B17, SGCZ, BICC1, DYNLL1, CLDN10, NDRG4, TLE2, WTIP and ARVCF*) located in 9 various chromosomes; (ii) significant CpG loci were again generally located in parts with various CpG density, but with a slight prevalence in shore regions and three cg in high-density regions in CpG island located in two various genes (*TLE2* and *DYNLL1*); (iii) main differences in CpG methylation levels between exposed and controls were detected in genes with similar relevance as the set of genes presented in Table 2, and additionally also to xenobiotic detoxification, cognitive functions or, for example, to type II diabetes.

## 3. Discussion

As mentioned previously, only one study focused on the quantitative analysis of DNA methylation in a real human population occupationally exposed to NP has been published [14]. However, no such report related to this type of exposure and qualitative DNA methylation approach by methylation chips exists. In this study, we tried to fill these gaps and also concentrate, besides the quantitative method, on the qualitative approach with the aim to evaluate both the effect of chronic long-term (years) and acute short-term (during monitoring day) exposure in the group of nanocomposite research workers. For this purpose, the Infinium MethylationEPIC BeadChips were used to identify the qualitative aspects of DNA methylation in more than 850,000 CpG loci distributed through all genes and intergenic regions. We particularly focused on the clarification of the knowledge related to the DNA methylation pattern modification/adaptation associated with NP exposure and the identification of particular changes in defined GpG loci and genes including their relevance.

Another advantage was the implementation of exposure monitoring including five PM fractions (ranking from <25 nm to 10 µm) in the workplaces in the study, as well as in our previous sampling periods. Changes in the processed samples induced by both processes (MAG welding in workshop 1, and grinding and milling in workshop 2) were implemented into this sampling when we compared sampling periods in 2016 [20,23] and 2018. Both processes were accompanied by generally different proportions of PM fractions, but differences in the processed materials and intensity of working processes in 2018 showed an increase in exposure to nano-fraction (<25–100 nm) related to both processes in comparison with 2016 (from 40.1% to 59.2% related to welding, and from 61.2% to 89.5% related to grinding and milling). Although the participants of the study were exposed in two workshops, we evaluated all the samples by both methylation assays, together with the aim to increase the validity of the obtained results and their interpretation.

Regarding both methodological approaches and the main information output related to acute and chronic exposure to NP, we obtained seemingly incoherent results. No effects of both exposures (acute and chronic) on the global methylation analysis by the quantitative approach were observed. Similarly, no effect of long-term chronic exposure analyzed by the qualitative approach using array chips was found. This discrepancy can easily be explained by the similar level of hypomethylated and hypermethylated CpG loci, which may lead to seemingly comparable global DNA methylation levels for the exposed and control groups. In the case of our study, 341 CpG loci were hypomethylated and 364 hypermethylated based on the microarray results. Noting that the used microarray chips contained a limited number of detectable CpG, we can presume that this hypo/hypermethylation balance was the main reason of discrepancy in results from both methodological approaches.

Another aspect is the interpretation of the effect of chronic exposure on the modification/adaptation of the DNA methylation pattern, as well as no effect of acute exposure. In our previous cytogenetic studies, we observed opposite effects related to the total frequency of micronuclei: no effect of chronic exposure and increased frequency of this type of DNA damage related to the increase of acute exposure [22,23]. The data from both studies perfectly match our previously published review study that focused on the effect of air pollution exposure, in which we also suggested the epigenetic model of adaptation to the environment [27]. According to this model, long-term exposure leads to the adaptation of the epigenome by DNA methylation modification, which impacts on the function of genes and consequently results in decreased levels of DNA damage. This is a crucial prerequisite for the survival of all organisms. In contrast to chronic exposure, acute exposure may result in a risk of no adequate adaptation (as in this study) or even a total lack of epigenetic modification in the case of a new exposure stressor. Despite the fact that the adaptation process is still not accepted by many genetic toxicologists, a lot of data supporting the epigenetic adaptation to the environment has accumulated, including the existence of epigenetic memory that plays a role in future exposure to the same stressor [27,28,29,30,31,32,33,34]. It is also important to note that the overall range of the differently methylated CpG sites caused by NP exposure in this study seems to be substantially lower in comparison with our previous study, which focused on the comparison of the DNA methylation pattern in children from two regions with substantially different air pollution levels. There we identified 9916 sites with significantly different methylation from a total of 27,578 analyzed CpG sites [26]. These results indicate the importance of time distribution of exposure: whereas in the air pollution study, the subjects were exposed permanently, the exposure to NP in the present study occurred only for a limited part of working days.

For the general concept of adaptation to the environment, the key aspect is the knowledge of particular changes related to various exposures. As demonstrated in Table 2, we identified the 14 most differentially methylated CpG loci after NP exposure, of which 9 are located in five protein coding genes, 3 others in one RNA coding gene and the 2 remaining in various intergenic regions. The comparison of these results, as well as the results summarized in Table 3 with previously published data, is generally difficult due to the novelty of these findings. Nevertheless, some studies investigating various other topics also identified modifications related to the genes identified in our study.

In our study, the most significant differences in the DNA methylation patterns were identified in *FGFR2* gene with the four most differentially methylated CpG loci. Additionally, another nineteen significant CpG in this gene were found. Hypomethylation was more frequent (three-fold difference) in comparison with hypermethylation. This may be explained by the higher probability of increased gene expression in NP exposed subjects. The protein encoding this gene (FGFR2) is a member of the fibroblast growth factor receptor family (FGFR), which is involved in a wide array of pathways known to play a significant role in cell proliferation and cancer including lung cancer. The effects of adverse environmental and lifestyle factors on respiratory allergies were recently studied in a small group of 10 volunteers by Illumina Methylation 450K BeadChip platform [35]. Similarly to our study, the authors confirmed differences in methylation sites in three genes including *FGFR2*. Moreover, changes in hypomethylation were also detected in the *FGFR2* gene involved in a network of nine respiratory system development genes, which also have connections with inflammatory signaling genes. Another, non-mammalian study analyzed the immunomodulatory effects of TiO_2_ NP in the sea urchin. It showed interactions with immune cells suppressing the expression of genes (including *FGFR2*) encoding for proteins involved in the immune response and apoptosis [36].

In relation to the other genes reported in Table 2, although limited information is available, some may be very interesting for our hypothesis on NP exposure effects. Firstly, changes in DNA methylation in *LGR6* gene were connected with the adaptation process to new tropical environmental conditions in Creole cattle [37]. This can be seen as a parallel with our finding related to adaptation to the environment after long-term exposure to NP. Another finding indicates that TMEM9B protein is a key component of inflammatory signaling pathways and suggests that endosomal or lysosomal components regulate these pathways [38]. Generally, *TMEM9B* gene is involved in an enhanced production of proinflammatory cytokines, which is also relevant to our study. Another gene, *FCGBP*, had significantly differently methylated three CpG loci from which two are located in an island region with the highest density of CpG. Interestingly, the differences in the function of this gene are associated with the possible changes in FEV1/FVC ratio (changes forced expiratory volume in 1 s/forced vital capacity analyzed by spirometry).

Other significant results summarized in Table 3 are related to DNA methylation differences between the exposed and control group in twelve additional genes. Among them, *TMEM18* gene seems to be mostly identified in various studies prevalently dealing with the topic of obesity, including adiposity, in relation to specific polymorphisms [39,40]. Single nucleotide polymorphisms (SNPs) were not analyzed in our study, but due to analyzed DNA methylation differences and no differences in BMI between the groups, we cannot exclude the effect of NP exposure on the metabolism of lipids driven by significant changes in DNA methylation. Moreover, we identified five more genes with relevance to this process (*TMEM9B*, *FCGBP*, *UGT2B15* and *16*, *SGCZ*, *WTIP*). This fact, as well as the results presented in a publication focused exclusively on the promoter methylation in *TMEM18* gene related to adiposity, may support our hypothesis [41]. In another study, differential gene expression analysis identified the deregulation of WTIP gene (analyzed also in our study) associated with BMI [42].

In relation to other significant results in the genes presented in Table 3, few relevant publications were recognized in PubMed database. *SHISA3* gene, hypermethylated in our study, was epigenetically inactivated in the colorectal cancer cell line [43]. A significant decrease of methylation in three specific CpG loci related to *UGTs* genes, involved in the detoxification of xenobiotics, can be interpreted as a direct reaction to NP exposure. One study identified the same CpG as we found in our study (cg07973162, cg07952421) with higher rates of methylation in Caucasians than in Asians. This result highlights the importance of ethnicity in xenobiotic sensitivity [44]. Another study demonstrates the existence of epigenetic memory by data showing the stability of epigenetic changes after a 22-week period in several genes including *BICC1* [45]. This suggests that a particular epigenetic modification can help the organism to better respond to future exposure to the stressor. The existence of epigenetic memory seems to be crucial in the process of adaptation to new conditions. *DYNLL1* gene was repeatedly linked with DNA repair processes, especially as an effector in non-homologous end joining (NHEJ) repair [46]. DNA methylation decrease in *DYNLL1* gene in a CpG island found in exposed subjects in our study could be the reason for intensive NHEJ repair processes in this group. An alteration in the mRNA expression of *CLDN10* gene, also identified in our epigenetic study, was found in female rats exposed to silver NPs [47]. We had already found the gender-related chromosomal aberration spectra changes in the NP exposed group [48]; thus, we cannot exclude the gender-related differences in epigenome aberrations related to NP exposure in the present study. However, due to a substantially lower number of females than males, it is impossible to obtain a relevant answer.

Based on the findings from the present study, we would like to briefly summarize some challenges for the future research. (i) It is generally important to accumulate more epigenetic data from large human studies investigating the effects of occupational NP exposure. (ii) Particular attention should be given to the effects of exposure time, gender, BMI, ethnicity and age of participants. (iii) The qualitative DNA methylation analysis methods should be preferred rather than quantitative methods. (iv) A detailed aerosol exposure monitoring of individual PM fractions including nano-fractions and their chemical compositions, even on a personal level, should accompany the results with the aim to better understand the association between the type of exposure and changes in DNA methylation patterns. (v) Long-term (few years) monitoring of epigenetic changes on an individual basis can also contribute to understanding the dynamicity of the epigenome modifications related to particular NP exposure, as well as to crucial changes in certain CpG of individual genes.

## 4. Materials and Methods

### 4.1. Study Population and Sampling

The study group was recruited from nanocomposite researchers from the same city and workplace. Apart from particulate matter (PM) fractions >100 nm, the study subjects were long-term (years) exposed to nano-fraction during their working activity. A total of 40 participants (20 exposed + 20 controls) were involved in the study, performed during the three-day period in September 2018. All were sampled twice per day (first: pre-shift, second: post-shift, or at a comparable time in the controls). The major route of exposure was by inhalation, as the exposed subjects did not use any respirators or other personal protective equipment. The general characteristics of the study subjects are presented in Table 4. The study population was composed of both genders (72.5% males + 27.5% females) with a relatively wide age range (21–72 years), but the exposed and controls did not significantly differ by age, gender or BMI. Although nine participants reported occasional smoking, the groups did not significantly differ in this parameter.

The exposed group was composed of long-term (14.5 ± 9.2 years) nanocomposite researchers. Detailed characteristics related to their exposure history, including common daily exposure and short-term exposure in the monitoring day, were obtained from questionnaires and are shown in Table 5. A significant increase of short-term exposure time in the monitoring day in comparison with common daily exposure (*p* = 0.028), allowed us to analyze the effect of short-term exposure. Moreover, detailed exposure monitoring data (including exposure to nano-fraction) related to working processes in two workshops and two background spaces on the day of monitoring are shown and described in Results.

A total of 80 whole venous blood samples were collected from both the exposed and control participants of the study at their workplace. All blood samples were thoroughly mixed with EDTA, stored at 4–10 °C and transported each day into a laboratory in Prague (approximately 110 km drive) for subsequent genomic DNA isolation.

All participants signed an informed consent form and had the opportunity to withdraw from participation at any time during the study, according to the Helsinki II declaration. The Ethical Committee of the General University Hospital in Prague and First Medical Faculty, Charles University approved the study (date of permission: 16 March 2017; registration mark: 2/17 Grant GA ČR—VFN).

### 4.2. Exposure Monitoring Measurements

On-line exposure monitoring related to both operations (MAG welding and machining (including grinding and milling)) during the shift and in two background spaces (basement and ground floor) included two standard aerosol spectrometers: (i) Scanning Mobility Particle Sizer (SMPS) (TSI Inc., SMPS 3936L, St. Paul, MN, USA); and (ii) Aerodynamic Particle Sizer (APS) (TSI Inc., APS 3321, St. Paul, MN, USA). The SMPS sized the particles according to their mobility in an electrostatic field and counted their number in individual size bins using a Condensation Particle Counter (CPC). In the CPC the particles were grown by condensation of working fluid to detectable size. The number of particles was detected by the laser light scattered on the particles. The size of the particles in the APS spectrometer was based on their inertial behavior when, after being accelerated, the aerosol particles passed two parallel laser beams perpendicular to the air flow, and the time of the passage of aerosol particle between the two beams was measured. The measured time was directly proportional to the particle size. Data from both SMPS and APS sizers were used to obtain more details related to size distribution and concentration of PM from the nanoscale range 6 nm up to 20 µm. More details related to exposure monitoring methods were published previously [20,49].

### 4.3. DNA Isolation and Quality Assessment

Genomic DNA (gDNA) from the all pre-shift and post-shift samples was extracted by the salting out procedure [50], from 5 mL whole venous blood collected into EDTA tubes. Isolated DNA samples were stored at a concentration of 50–500 µg/µL at −20 °C. The concentration and quality of DNA were controlled using the Nanodrop ND-1000 spectrophotometer before DNA methylation analysis.

### 4.4. Quantitative DNA Methylation Analysis

The content of 5-methylcytosine (5-mC) was assessed in triplicate in all 80 gDNA samples using 5-mC DNA ELISA Kit (Zymo Research, Irvine, CA, USA) following the manufacturer’s instructions. Briefly, the indirect ELISA technique was used in the workflow. Denatured, single-strand DNA samples (100 ng DNA/well) were added to the wells in 5-mC coating buffer. Anti-5-methyl cytosine monoclonal antibody and horseradish peroxidase (HRP) conjugated secondary antibody were prepared in 5-mC ELISA buffer and added to the wells. Detection of 5-mC, which occurs after the addition of HRP developer, was measured by using the ELISA plate reader at absorbance 405 nm. Final results were expressed as % 5-mC/total cytosine content.

### 4.5. Qualitative Infinium HD Methylation Assay

The array-based methodological approach was used for the advanced methylation assay. Infinium MethylationEPIC BeadChips (Illumina, San Diego, CA, USA), allowing interrogation of more than 850,000 CpG loci dispersed through the whole human genome, were used in this study. Individual methodological steps were as follows: (i) A total of 500 ng gDNA was treated overnight with sodium bisulfite using Zymo EZ DNA Methylation^TM^ Kit (Zymo Research, Irvine, CA, USA) for the conversion of unmethylated cytosines to uraciles, while methylated cytosines remain unchanged. (ii) Bisulfite-converted gDNA was processed according to the manufacturer’s protocol (Infinium^®^ HD Assay Methylation for Methylation Protocol Guide #15019519v01 from October 2015 provided by Illumina) including the enzymatic fragmentation, precipitation, resuspension and overnight hybridization followed by washing and BeadChip staining. (iii) All chips were scanned by iScan System (Illumina, San Diego, CA, USA) for final imaging. The methylation status at each CpG site was estimated by measuring the intensity of the pair of methylated and unmethylated probes. For the Quality Assurance/Quality Control assessment (QA/QC) the three random samples were used as replicates on various chips. The correlation coefficient between replicates was high (Pearson’s correlation coefficient *r* = 0.999, *p* < 0.001).

### 4.6. Statistical Analysis

Basic descriptive statistics (mean, standard deviation, median, minimum, maximum), including t-test for normally distributed variables, were calculated using Microsoft Excel 2013.

All advanced statistical analyses related to qualitative methylation levels in individual CpG loci were processed using scripting in the R environment. Specifically, (i) raw microarray data were downloaded as idat files, (ii) imported to the R program, and (iii) processed by minfi package in Bioconductor [51]. Data were normalized using implemented quantile method and background correction. A series of filtering on methylation probes were consecutively performed with the aim to exclude (i) probes with results under the limit of detection, (ii) probes related to gonosomes CpG sites, (iii) non-specific probes in SNPs sites, and (iv) probes that have shown to be cross-reactive. A list of non-specific CpG sites was previously published [52] and imported to the R script as a csv table. Finally, a total of 99,660 CpG sites were excluded from subsequent analysis.

Beta values for the determination of the level of methylation defined as the ratio of the fluorescent signals from the methylated vs. unmethylated sites were calculated using this package. Significant beta values < 0.2 or > 0.7 were considered as hypomethylation or hypermethylation, respectively, as reported previously [26]. Preprocessing analyses were performed to study the distribution of beta values and the variation of methylation across all samples. Principal component analysis (PCA) was performed to identify directions of maximum variance using a covariance matrix. This approach was applied to assess the potential effect of study groups (exposed and control, pre-shift and post-shift). The batch effect of microarrays was not observed in PCA as variance cluster on filtered data.

A subsequent analysis, linear model in limma package, including other covariates as age and gender, was performed according to the published protocol [53]. Furthermore, the contrast matrix in the linear model was applied to detect exposure-specific differentially methylated sites.

The *p*-values were adjusted for multiple testing using the Benjamini–Hochberg methods (BH, *p*-value adjusted) [54], which control the expected false discovery rate (FDR) below the specified value.

## 5. Conclusions

To the best of our knowledge, this is the first human biomonitoring study focused on the investigation of DNA methylation changes in both long-term and short-term NP exposed subjects, analyzed by the qualitative array approach. The obtained results showed the ability of the human body to modify the epigenome, by both hypomethylation and hypermethylation of specific CpGs related to this type of exposure. This epigenetic adaptation was observed after long-term chronic exposure, but not following short-term acute exposure. Significantly differently modified CpGs were associated with lipid metabolisms, immune system, lung functions, signaling pathways, cancer disease development and xenobiotic detoxification. These data are important for the interpretation of the biological effects of NP, including the possible risk of disease, in later life.

This study also opens numerous new questions for future research not only in relation to the biological safety of NP, but also to a link between NP exposure and lipid metabolism or repair processes. Moreover, even though we have presented the ability of the human body to modify the function of genes to the NP exposure, and the crucial long-term biological effects leading to the development of a particular disease were indicated, there is a demand for more human studies to continue to fully understand the health risks of this exposure.

## Figures and Tables

**Table 1:** Total number concentrations of five PM fractions (nano to 10 µm) per cm^3^ measured by on-line monitoring (SMPS and APS) during the shift related to the individual working processes including backgrounds.

	**Total Number Concentrations of PM Fractions per cm^3^**					
Processes	1.	2.	3.	4.	5.	Total
Backgrounds	<25 nm	25–100 nm	100 nm–1 µm	1−2.5 µm	2.5−10 µm	<10 µm
MAG welding	1680	3840	3790	1.1	0.215	9311
Grinding and Milling	9700	16400	3040	16	2.53	29159
Background, basement	574	6260	2370	0.42	0.078	9204
Background, ground floor	92.5	3550	1680	0.232	0.006	5323

Abbreviations: SMPS, scanning mobility particle sizer; APS; aerodynamic particle sizer; MAG, metal active gas.

**Table 2:** Overview of information related to the 14 most differentially methylated CpG loci (bold) in the exposed group when compared to the controls including all other significant results in the same gene.

# Chrom.	CpG Locus (cg number)	Island Relation	Gene	Relevance or Phenotype	log FC	*p*-Value	*p*-Value Adjusted
#1	**cg04811114**	Open Sea	*LGR6*	Signaling pathways	1.645	1.30 × 10^−7^	0.002
	**cg06825163**	Open Sea	“	Breast carcinoma	1.647	2.20 × 10^−7^	0.003
	cg00588342	Open Sea	“		0.294	1.44 × 10^−5^	0.027
	cg26347746	Open Sea	“		0.863	2.61 × 10^−7^	0.003
	cg25270774	Open Sea	“		1.233	3.25 × 10^−7^	0.003
	cg05044291	Open Sea	“		1.282	3.11 × 10^−7^	0.003
#6	**cg12771717**	N Shore	HCG27*	Regulation	−1.813	1.77 × 10^−6^	0.009
	**cg03030317**	N Shore	“	Ankylosing	−1.699	2.34 × 10^−7^	0.003
	**cg09622121**	N Shore	“	spondylitis	−1.651	3.21 × 10^−7^	0.003
	cg23595396	N Shore	“	Alopecia	−1.059	7.77 × 10^−7^	0.006
	cg24023453	N Shore	“	Asthma	−0.833	1.24 × 10^−6^	0.008
				Lung carcinoma			
				Blood cells count			
#7	**cg18467790**	N Shelf	*RADIL*	Hypothyroidism	3.111	3.97 × 10^−8^	0.001
#10	**cg25052156**	N Shore	*FGFR2*	Signaling pathways	−1.584	4.21 × 10^−9^	<0.001
	**cg06791446**	N Shore	“	Kinase activity	−1.579	3.42 × 10^−8^	0.001
	**cg10379346**	N Shore	“	Reg. cell prolif.	−1.558	4.18 × 10^−8^	0.001
	**cg16653991**	Open Sea	“	Apoptosis	1.659	7.81 × 10^−8^	0.001
	cg11430259	N Shore	“	Lung carcinoma	−1.442	3.74 × 10^−7^	0.004
	cg02210151	N Shore	“	Brest carcinoma	−1.152	4.66 × 10^−8^	0.001
	cg22633036	N Shore	“	Colorectal cancer	−1.117	2.63 × 10^−7^	0.003
	cg13437682	N Shore	“	Blood pressure	−1.025	1.99 × 10^−8^	<0.001
	cg16961769	Open Sea	“		−0.843	1.41 × 10^−6^	0.008
	cg12669518	Open Sea	“		−0.820	1.11 × 10^−7^	0.002
	cg17681491	N Shore	“		−0.791	1.35 × 10^−8^	<0.001
	cg14968358	Open Sea	“		−0732	3.37 × 10^−5^	0.043
	cg23248910	Open Sea	“		−0.719	3.71 × 10^−7^	0.003
	cg13707729	Open Sea	“		−0.486	1.02 × 10^−5^	0.023
	cg17723924	Open Sea	“		−0.405	5.75 × 10^−6^	0.017
	cg25833171	Open Sea	“		−0.351	2.71 × 10^−5^	0.038
	cg12990750	Open Sea	“		−0.317	4.31 × 10^−5^	0.048
	cg07344086	Open Sea	“		−0.261	2.53 × 10^−5^	0.037
	cg25409939	Open Sea	“		0.627	6.14 × 10^−6^	0.018
	cg03552039	Open Sea	“		0.634	3.85 × 10^−7^	0.003
	cg17280705	Open Sea	“		0.638	1.37 × 10^−5^	0.027
	cg08195415	Open Sea	“		0.665	5.48 × 10^−6^	0.017
	cg08899523	Open Sea	“		1.452	6.86 × 10^−8^	0.001
	**cg07044115**	Open Sea	out		−1.778	1.07 × 10^−8^	<0.001
#11	**cg15570860**	S Shore	*TMEM9B*	Signaling pathways	−3.843	1.55 × 10^−7^	0.002
	cg16733419	N Shelf	“	Proinf. cytokines↑	−0.339	2.25 × 10^−5^	0.034
				Hemoglobin level			
				BMI			
#19	**cg03635532**	CpG Island	*FCGBP*	Lung function	2.373	7.94 × 10^−10^	<0.001
	cg18588295	CpG Island	“	Triglyceride change	0.317	8.07 × 10^−6^	0.021
	cg08054032	S Shore	“		0.557	1.10 × 10^−8^	<0.001
	**cg14764203**	Open Sea	out		1.530	5.99 × 10^−6^	0.018

Abbreviations: *LGR6*, Leucine-rich repeat containing G; *HCG27*, HLA complex group 27 (* RNA gene); *RADIL*, Rap associating with DIL domain; *FGFR2*, Fibroblast growth factor receptor 2; *TMEM9B*, TMEM9 domain family member B; *FCGBP*, Fc fragment of IgG binding protein; out, CpG locus is out of gene.

**Table 3:** Overview of information related to other significant results in the exposed group when compared with the controls with 3 or more differently methylated CpG loci in the same gene.

# Chrom.	CpG Locus (cg number)	Island Relation	Gene	Relevance or Phenotype	log FC	*p*-Value	*p*-Value Adjusted
#2	cg18049933	N Shore	*LOC100996579*	uncharacterized	0.588	4.20 × 10^−5^	0.048
	cg15237618	N Shore	“		0.658	1.97 × 10^−5^	0.032
	cg23987493	N Shore	“		0.671	7.68 × 10^−6^	0.021
	cg17611880	N Shore	*TMEM18*	Transcription rec.	0.473	3.89 × 10^−5^	0.046
	cg18263335	N Shore	“	BMI	0.852	2.37 × 10^−6^	0.012
	cg27237671	N Shore	“	Body fat distrib.	0.863	7.28 × 10^−6^	0.020
#4	cg22541001	S Shore	*SHISA3*	Signaling pathways	0.370	3.87 × 10^−7^	0.004
	cg13587180	S Shelf	“	Tumor suppressor	0.448	1.12 × 10^−6^	0.007
	cg11065575	S Shelf	“	Cytokine level	0.541	4.30 × 10^−5^	0.048
				Type II diabetes			
	cg13365324	Open Sea	*UGT2B15*	Blood cell distrib.	1.243	5.61 × 10^−6^	0.018
	cg07973162	Open Sea	*and B17*	Cholesterol	−1.171	7.00 × 10^−6^	0.020
	cg07952421	Open Sea	“	Triglyceride	−1.103	1.88 × 10^−5^	0.032
				Xenobiotics detox.			
#8	cg27405903	Open Sea	*SGCZ*	Cognitive function	0.457	3.36 × 10^−6^	0.014
	cg05986192	Open Sea	“	BMI	0.641	2.07 × 10^−6^	0.011
	cg17481116	Open Sea	“		0.785	1.37 × 10^−6^	0.008
#10	cg08466030	Open Sea	*BICC1*	Gen expr. regul.	0.397	2.70 × 10^−5^	0.038
	cg27040468	Open Sea	“	Signaling pathways	0.660	1.07 × 10^−6^	0.007
	cg12342675	Open Sea	“	Uric acid level	0.987	8.73 × 10^−7^	0.006
#12	cg27279351	CpG Island	*DYNLL1*	Intrac. transport	−0.305	1.62 × 10^−7^	0.002
	cg19946631	N Shore	“	Cellular senescence	−0.299	4.68 × 10^−6^	0.016
	cg25284772	N Shore	“	Reticulocyte count	−0.241	1.24 × 10^−6^	0.008
				Blood pressure			
#13	cg24545961	S Shore	*CLDN10*	Signaling pathways	−1.498	5.45 × 10^−8^	0.001
	cg25702335	S Shore	“		−1.488	1.14 × 10^−7^	0.002
	cg24529736	S Shore	“		−0.801	1.26 × 10^−7^	0.002
	cg05709657	S Shore	“		−0.741	1.07 × 10^−5^	0.023
#16	cg04484415	N Shore	*NDRG4*	Signaling pathways	0.541	4.28 × 10^−5^	0.048
	cg05725404	N Shore	“	Apoptosis	0.714	1.10 × 10^−5^	0.024
	cg17457090	N Shore	“	QT interval	0.616	3.47 × 10^−5^	0.043
				Colorectal cancer			
#19	cg00857137	CpG Island	*TLE2*	Signaling pathways	0.377	4.87 × 10^−6^	0.017
	cg26717563	N Shore	“	Blood cells count	0.472	2.64 × 10^−6^	0.012
	cg19334452	CpG Island	“		0.533	1.66 × 10^−6^	0.009
	cg11374335	N Shore	*WTIP*	Cellular senescence	0.383	2.90 × 10^−11^	<0.001
	cg06177396	N Shore	“	Transcr. regulator	0.408	8.65 × 10^−12^	<0.001
	cg04928251	N Shore	“	Metal ion binding	0.547	1.00 × 10^−10^	<0.001
			“	BMI			
				Triglyceride			
				Blood cells count			
#22	cg07821417	N Shelf	*ARVCF*	Blood cells count	0.317	2.00 × 10^−5^	0.032
	cg16324072	S Shelf	“	Blood metab. level	0.337	4.72 × 10^−6^	0.016
	cg13823643	S Shore	“	Serum metab. level	0.373	6.16 × 10^−7^	0.005

Abbreviations: *LOC100996579*, “uncharacterized gene”; *TMEM18*, Transmembrane protein 18; *SHISA3*, Shisa family member 3; *UGT2B15/B17*, UDP glucuronosyltransferase family 2 member B15/B17; *SGCZ*, Sarcoglycan zeta; *BICC1*, BicC family RNA binding protein 1; *DYNLL1*, Dynein light chain LC8 type 1; *CLDN10*, Claudin 10; *NDRG4*, NDRG family member 4; *TLE2*, TLE family member 2, transcriptional corepressor; *WTIP*, WT1 interacting protein; *ARVCF*, ARVCF delta catenin family member.

**Table 4:** General characteristics of the exposed and control subjects.

Characteristics Group	N	Mean ± SD	Median (Range)	*p*
Age (years)				
All	40	42.1 ± 11.9	41 (21−72)	
Exposed	20	39.3 ± 11	36.5 (24−65)	0.129
Controls	20	45.0 ± 12.4	46 (21−72)	
Gender (M/F)				
All	29/11	N/A	N/A	
Exposed	14/6	N/A	N/A	0.731
Controls	15/5	N/A	N/A	
BMI (kg/m^2^)				
All	40	26.4 ± 5.1	26 (19−38.9)	
Exposed	20	26.8 ± 5.3	26.4 (19−36.7)	0.655
Controls	20	26 ± 5	24.9 (20.2−38.9)	

Abbreviations: N, number of subjects; SD, standard deviation; M, males; F, females; N/A, not applicable; BMI, body mass index.

**Table 5:** Exposure characteristics obtained from questionnaires.

Characteristics Group	Mean ± SD	Median (Range)	*p*
NP exposure record			
Exposed (*n* = 20)			
Long-term (years)	14.5 ± 9.2	12 (3−32)	
Common daily (min)	115.5 ± 68.3	105 (60−270)	0.028
Short-term (min)	154.5 ± 34.1	150 (120−240)	

Abbreviations: N, number of subjects; SD, standard deviation; *p*, comparison of common daily exposure (chronic) and short-term exposure in monitoring day (acute).

## Abbreviations

16HBE14o-	Human bronchial epithelial cells
5-mC	5-methyl-cytosine
APS	Aerodynamic particle sizer
*ARVCF*	ARVCF delta catenin family member
BEAS-2B	Human bronchial epithelial lung cells
BH	Benjamini-Hochberg methods
*BICC1*	BicC family RNA binding protein 1
BMI	Body mass index
*CLDN10*	Claudin 10
CPC	Condensation particle counter
DNMT	DNA methyltransferase
*DYNLL1*	Dynein light chain LC8 – type 1
FDR	False discovery rate
*FCGBP*	Fc fragment of IgG binding protein
FEV1	Forced expiratory volume in 1 s
*FGFR2*	Fibroblast growth factor receptor 2
FVC	Forced vital capacity
HaCaT	Human keratinocytes cells
HepG2	Human liver cancer cells
*HCG27*	HLA complex group 27
HRP	Horseradish peroxidase
ITO	Indium tin oxide
*LGR6*	Leucine rich repeat containing G
MAG	Metal active gas
MN	Micronuclei
MRC5	Human fetal lung cells
MWCNT	Multi walled carbon nanotubes
*NDRG4*	NDRG family member 4
NHEJ	Non-homologous end joining repair
NM	Nanomaterials
NP	Nanoparticles
PCA	Principal component analysis
PM	Particulate matter
*RADIL*	Rap associating with DIL domain
*SGCZ*	Sarcoglycan zeta
*SHISA3*	Shisa family member 3
SMPS	Scanning mobility particle sizer
SNPs	Single nucleotide polymorphisms
SPION	Supermagnetic iron nanoparticles
SWCNT	Single walled carbon nanotubes
*TLE2*	TLE family member 2, transcriptional corepressor
*TMEM18*	Transmembrane protein 18
*TMEM9B*	TMEM9 domain family member B
TSS	Transcription start site
*UGT2B15/B17*	UDP glucuronosyltransferase family 2 member B15/B17
WGBS	Whole-genome bisulfite sequencing
*WTIP*	WT1 interacting protein
